# Rupturing Abdominal Aneurysm Presenting as Acute Coronary Syndrome

**DOI:** 10.7759/cureus.9296

**Published:** 2020-07-20

**Authors:** Willeke Van der Stuijt, Remko S Kuipers

**Affiliations:** 1 Cardiology, Amsterdam University Medical Centers, Amsterdam, NLD; 2 Heart Center, Onze Lieve Vrouwe Gasthuis, Amsterdam, NLD

**Keywords:** acute coronary syndrome (acs), acute abdominal aneurysm, pseudo myocardial infarction, cardiac troponin, non-st elevation myocardial infraction, non st-segment elevation myocardial infarction

## Abstract

A 61-year-old male presented to the emergency ward with pain in his upper abdomen. Due to an abnormal electrocardiogram (ECG) and elevated cardiac enzymes the cardiologist was consulted to exclude cardiac pathology. The consulting cardiologist advised to exclude an abdominal diagnosis before treating the condition as an acute coronary syndrome (ACS). Before noninvasive imaging had been performed, the clinical situation deteriorated and an emergency laparotomy revealed a ruptured aortic aneurysm. Despite immediate revascularization multiple organ failure ensued and the patient died a few days later. This case illustrates that the suspicion of ACS should never delay the investigation of other life-threatening disorders. Contrarily angina, ECG abnormalities, and myocardial ischemia are all well known to concur with major vascular, intra-abdominal, intra-cranial, and pulmonary pathology; hence these other life-threatening conditions should always be considered and preferably be ruled out prior to further investigation and treatment of ACS.

## Introduction

The leading symptom that initiates the diagnostic and therapeutic cascade in patients with suspected acute coronary syndrome (ACS) is chest pain. Typical chest pain is characterized by a retrosternal sensation of pressure or heaviness (‘angina pectoris’), while atypical presentations include epigastric pain, indigestion-like symptoms, and even isolated dyspnoea [1]. Depending on the concurrent observations during electrocardiography (ECG) and the results from laboratory analyses, ACS can be subdivided into ST-elevation ACS and non-ST-elevation ACS (NSTE-ACS). ST-elevation ACS is characterized by persistent (>20 min) ST-segment elevation on the ECG. In NSTE-ACS several (other) types of ECG abnormalities can be observed. NSTE-ACS can be further differentiated into NSTE-myocardial infarction (NSTE-MI) and unstable angina pectoris (UAP), depending on whether or not, respectively, cardiomyocyte necrosis is demonstrated at the myocardial level by measurement of cardiac muscle enzymes (creatine kinase myocardial band; CK-MB) or, more sensitively, cardiac troponins [1].

If an ACS is suspected, the adagium ‘time is (cardiac) muscle’ accounts and immediate measures are taken to restore the balance between myocardial oxygen supply and demand within the coronary arteries, e.g. by reducing heart rate, blood pressure, preload and myocardial contractility to decrease myocardial oxygen demand, and by the administration of platelet aggregation inhibitors and anticoagulants or by percutaneous coronary intervention (PCI) to restore oxygen supply through removal of intraluminal thrombi [1].

However, next to myocardial ischemia, several other cardiac and noncardiac conditions are known to cause acute (a)typical chest pain, ECG abnormalities, elevation of cardiac enzymes/proteins, and even all of these together. More importantly, the proper treatment of each of these conditions -- that might present quite similar -- might in fact be opposite, such as the administration of anticoagulation in ACS but not during hemorrhagic shock.

Hence, to prevent patients experiencing unnecessary delay, invasive diagnostics or contra-indicated medical regimens, it is extremely valuable for every clinician to be aware of the variety of causes that can cause chest pain, ECG changes, and/or cardiomyocyte necrosis. In this case report, we present a patient who was referred to our ED with acute epigastric pain, ECG abnormalities, and a rise of troponin levels.

## Case presentation

A 61-year-old man was brought to our emergency ward presenting with upper abdominal pain, radiating from his lower abdomen and to his back. He was sweating profusely. His blood pressure and heart rate at presentation were respectively 97/70 mmHg and 98 beats/min and these values remained stable. His medical history includes hypertension, gout, chronic obstructive pulmonary disease (COPD) stage Gold II, obstructive sleep apnea syndrome (OSAS), and a recent polypectomy from his colon. He uses no medication. His current weight is 110 kg and he has smoked one package of cigarettes every day for at least 30 years.

**Figure 1:**
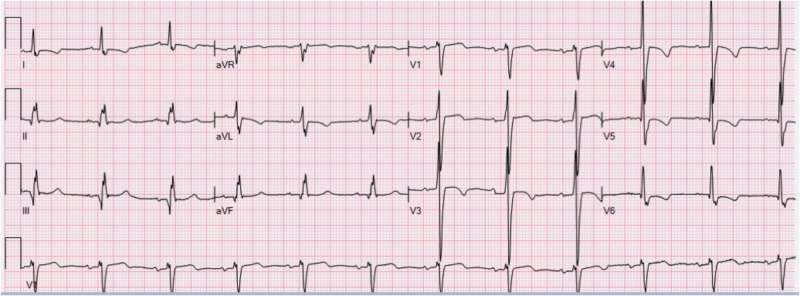
The ECG of the patient with a rupturing abdominal aneurysm. ECG, electrocardiogram

Upon presentation he recalls having had vague abdominal pain since the abdominal intervention (polypectomy) three weeks ago. The acute increase in pain had begun this early morning and after waiting for several hours he had decided to call an ambulance. The pain has lasted for six hours now. A second survey revealed a blood pressure of 106/71 mmHg and a heart rate of 71 beats/min. His temperature was 36.2°C, his breathing frequency 15/min, and his oxygen saturation (SpO2) 95%. Physical examination revealed an obese man with normal peristalsis. There was no change in pain upon palpation. The laboratory results for his blood count, liver and kidney functions were normal, with the exception of an elevated leukocyte count (16x10^9/L), C-reactive protein (CRP) (67 mg/L) and high sensitive (HS)-troponin I, which is 35 ng/L. The ECG showed negative T-waves in I, aVL, V3-V6, while his previous ECG from a previous admission was completely normal (Figure 1). In the next hours his condition remained stable and abdominal echography was awaited. In the meantime, the cardiology department is consulted because of the deviant ECG and the release of cardiac enzymes to exclude an ACS as the cause of his abdominal pain. The consulting cardiologist advised to exclude an abdominal diagnosis before treating his condition as an ACS. HS-troponin I levels were repeated after three hours and have risen to 65 ng/L.

Six hours after admission his condition deteriorated. The abdominal pain increased, while his blood pressure dropped to 83/56 mmHg. The consulting ICU physician suspected gastrointestinal perforation or bleeding and an emergency CT scan was ordered. However, due to the unstable condition, the treating surgeon decided to perform an emergency laparotomy. This showed a ruptured abdominal aneurysm and a prosthesis was placed. Additionally an embolectomy from his left leg was performed. The patient returned to the ICU department for respiratory and hemodynamic support. Within the next hours the patient remained anuric and lactate continued to rise despite inotropic support. During the night multiple organs began to fail and another emergency laparotomy revealed gastrointestinal ischemia for which a sigmoid resection was performed and a stoma was placed. Despite all this support the patient died the following day from systemic inflammatory response syndrome secondary to multi-organ failure after severe blood loss and a substantial period of hypotension as a consequence of the ruptured aneurysm. 

## Discussion

Typical and atypical angina, accompanied by ECG abnormalities with or without a (significant) rise in cardiac enzymes might result from both cardiac and noncardiac conditions. It is important to discern these two, as for example, treating a suspected ACS with anticoagulation on top of double platelet aggregation inhibitors might worsen the prognosis of a patient who presents with either a vascular dissection or aneurysm.

The ECG abnormalities have been noted in several conditions besides ACS. In the absence of chest pain, examples of such conditions that may cause reversible and irreversible changes of the ECG, are: different cardiomyopathies, athletic heart syndrome, congenital coronary artery-ventricular multiple micro-fistulas, cardiac metastasis, cardiac sarcoidosis, cardiac memory, antiarrhythmic drug effects, cocaine use, pulmonary edema, and intra-cerebral pathologies [2]. In the presence of pain, either typically located on the chest, but atypically sometimes located in the neck, arms, abdomen or even in the groin or legs, several life-threatening conditions might explain -- mostly transient -- abnormalities in the ECG. These could be from either a cardiac or noncardiac origin. Conditions that do concern the heart, such as pericarditis and myocarditis, should be differentiated from an ACS by anamnesis, echocardiographic findings, or even coronary angiography. A second group includes conditions that cause ECG abnormalities and a rise in troponin levels as a consequence of the so-called demand (i.e. type 2) myocardial ischemia. In fact, ST-elevation has been seen in 3.4%-24% of patients classified as having type 2 ischemia, while 22%-72% of these patients showed no evidence of concomitant coronary artery disease during subsequently performed coronary angiographies [3]. Causes of demand ischemia are severe sepsis, perforated ulcers, gastro-enteritis, cholecystitis, and pancreatitis [4-6]. Also hypertensive emergencies, aortic dissection, pulmonary embolism and arguably the acute stress syndrome known as Takotsubo cardiomyopathy are included in this group [2, 7-10]. In case of abdominal pain, intra-abdominal pathology has been postulated to trigger ECG abnormalities by a cardio-biliary reflex [4, 11-13]. It has been hypothesized that this vagally mediated reflex causes T-wave changes secondary to abdominal stimulation, such as gastro-intestinal hemorrhage, cholecystitis and pancreatitis, but also in case of intracranial bleeds. However, it could also be argued that these latter conditions in fact cause ECG changes or a rise in troponin levels due to demand ischemia secondary to the adrenalin release caused by the primary problem.

Similar to life-threatening coronary and other cardiac causes of ECG abnormalities, life-threatening noncardiac causes need to be ruled out as soon as possible to reduce patient morbidity and mortality. Moreover, rushed decision-making could result in improper medical interventions and invasive procedures which could culminate in increased patient morbidity and mortality. 

Taken together, a rupturing abdominal aneurysm may mimic atypical coronary syndrome with regard to pain, electrocardiographic abnormalities, and a rise-and-fall in cardiac enzymes. However, the suspicion of an ACS in a hemodynamically unstable patient should not defer further investigation of other life-threatening conditions. On the contrary, as correlations between major vascular, intra-abdominal, intra-cranial, pulmonary pathology and electrocardiographic abnormalities are well known, such conditions should always be considered and preferably ruled out prior to further treatment of ACS. 

## Conclusions

Several life-threatening conditions present with acute chest or abdominal pain. Correlations between major vascular, intra-abdominal, intra-cranial, pulmonary pathology, and ECG abnormalities are well known. Life-threatening conditions often coincide with a heavy cardiac workload, resulting in the myocardial ischemia and a rise of cardiac enzymes. Hence, other life-threatening conditions that mimic an ACS should be considered and preferably be ruled out prior to further investigation and/or treatment of a suspected myocardial infarction. A rupturing aortic aneurysm may cause angina, ECG changes, and elevation of cardiac enzymes prior to clinical signs of hemodynamic deterioration.
